# Association Between Sarcopenia and Buttock Pain Among Middle-Aged and Older Chinese People: Evidence from the China Health and Retirement Longitudinal Study

**DOI:** 10.3390/healthcare13111311

**Published:** 2025-05-31

**Authors:** Jian Jin, Huibin Long, Huiwen Zhang, Chuanhui Zhang, Jianhao Lin

**Affiliations:** 1 Arthritis Clinical and Research Center, Peking University People’s Hospital, No.11 Xizhimen South Street, Beijing 100044, China; bjmujin@bjmu.edu.cn (J.J.); longhuibin@pkuph.edu.cn (H.L.); 2162049620@pku.edu.cn (H.Z.); 2 Department of Joint and Sports Medicine, Chaoyang Central Hospital, No. 6 Section 2 Chaoyang Avenue, Shuangta District, Chaoyang 122000, China; zhang_chuanhui@hotmail.com

**Keywords:** sarcopenia, buttock pain, muscle mass, CHARLS

## Abstract

Background: Sarcopenia and buttock pain are highly prevalent in older adults and exert profound negative effects on quality of life. Little is known about the association between sarcopenia and buttock pain. Methods: This study performed cross-sectional and longitudinal analyses based on prospective cohort study data from the 2015 and 2020 waves of the China Health and Retirement Longitudinal Study (CHARLS). A total of 12,884 community-dwelling adults aged ≥45 years were included in the cross-sectional analysis, and 10,511 of these participants, free of buttock pain at baseline, were further investigated to assess incident buttock pain. Sarcopenia status was categorized as non-sarcopenia, possible sarcopenia, and sarcopenia according to the 2019 Asian Working Group for Sarcopenia and the 2021 Chinese consensus criteria. Logistic regression models adjusted for sociodemographic and health-related covariates were performed to estimate associations between sarcopenia status and buttock pain. Results: After adjusting for covariates, possible sarcopenia, but not sarcopenia, was associated with prevalent buttock pain (OR 1.23, 95% CI 1.03–1.48). After 5 years of follow-up, participants with sarcopenia were more likely to develop incident buttock pain (OR 1.37, 95% CI 1.03–1.81). Among sarcopenia components, poor physical performance was linked to prevalent pain (OR 1.25, 95% CI 1.05–1.50) and low handgrip strength predicted incident pain in males (OR 1.31, 95% CI 1.07–1.60). Appendicular muscle mass was not independently associated with either prevalent or incident buttock pain. Conclusions: In middle-aged and older Chinese adults, sarcopenia is an independent risk factor for incident buttock pain. Early screening and interventions of sarcopenia may help to mitigate the burden of buttock pain and its associated disability.

## 1. Introduction

Sarcopenia is defined as a progressive, generalized skeletal muscle disorder characterized by an accelerated decline in muscle mass and function [1]. Its global prevalence is estimated at 10–22%, varying according to the diagnostic criteria [2,3,4]. Sarcopenia is an established risk factor for falls, fractures, metabolic derangements, and disability in older adults, and it correlates with a spectrum of adverse outcomes—including increased postoperative complications, prolonged hospitalization, and elevated mortality [1,4,5]. Recent studies have further implicated sarcopenia as a risk factor in the development and progression of chronic conditions such as cardiovascular disease [6], respiratory disorders [7], psychiatric illness [8], and cognitive impairment [9]. Collectively, these comorbidities and resultant disabilities contribute to diminished quality of life, loss of independence, and heavy burden on healthcare systems [7]. Consequently, sarcopenia represents a critical and growing public health challenge.

Musculoskeletal pain disorders rank among the leading causes of global disability [10]. Chronic musculoskeletal pain has been one of the most significant sources of health loss worldwide for its psychological sequelae, economic burden, and decline in quality of life [11,12]. Buttock pain is one of the most common types of musculoskeletal pain and a frequent complaint encountered in clinical practice [13]. Possible etiologies of buttock pain include dynamic impingement of peri-articular structures, entrapment neuropathies, myalgia and myofascial pain syndromes, gluteal tendinopathies, and other less common causes [13,14,15]. Buttock pain can substantially impair patients’ quality of life by limiting mobility, reducing participation in daily activities, disrupting sleep, and adversely affecting psychological well-being. Identifying modifiable risk factors would help to alleviate the disease burden of buttock pain.

Previous investigations into the epidemiology of buttock pain remain limited, possibly owing to the close anatomical relationship between the hip and gluteal regions. In many studies, symptoms originating in the buttock are subsumed under the broader category of hip pain and are infrequently reported as an independent entity [16]. Previous studies on the relationship between sarcopenia and buttock or hip pain remain limited, with most focusing on isolated muscle characteristics, such as muscle mass, muscle strength, or biomechanical factors. One study has demonstrated that prolonged sitting, poor posture, and physical inactivity are significantly associated with an increased risk of buttock pain [17]. Research exploring the association between muscle mass and hip pain has reported that individuals with hip pain often exhibit reduced peri-articular muscle mass and diminished muscle strength [18,19,20]. Moreover, based on bidirectional Mendelian randomization, one study demonstrated a causal relationship between appendicular lean mass, handgrip strength, gait speed, and osteoarthritis, further showing that reduced muscle mass is associated with an increased risk of osteoarthritis [21]. However, other investigations have failed to confirm a consistent association between muscle mass and hip pain [22,23,24]. Skeletal muscles play a critical role in stabilizing hip structures and modulating joint load distribution, potentially influencing the pathogenesis of degenerative disorders [25,26]. The association between muscle characteristics and buttock pain requires further investigation.

In this study, we conducted cross-sectional and longitudinal analysis based on the China Health and Retirement Longitudinal Study (CHARLS). In the cross-sectional study, we assessed the association between sarcopenia and the prevalence of buttock pain. In the longitudinal study, we examined how baseline sarcopenia status predicted the subsequent incidence of buttock pain over follow-up. Considering that the three components of sarcopenia (i.e., muscle mass, strength, and physical performance) reflect different aspects of muscle health and may play different roles in the pathogenesis and development of buttock pain, we investigated the contributions of individual sarcopenia components to buttock pain outcomes.

## 2. Materials and Methods

### 2.1. Study Population

CHARLS is a nationally representative longitudinal survey initiated in 2011 in China. High-quality data were collected via personal interview using structured questionnaires. Under the strategy of probability proportional to size, CHARLS employed a four-stage stratified random sampling process at the county, neighborhood, household, and respondent levels to select eligible participants. In the county-level sampling, with the exception of Hainan, Ningxia, Taiwan, Hong Kong, Macau, and Tibet, all counties were stratified by region, urban–rural status, and gross domestic product (GDP) per capita, and 150 representative counties from 28 provinces were selected. For neighborhood-level sampling, rural administrative villages and urban communities were designated as the primary sampling units (PSUs), and three PSUs were randomly selected per county. During household sampling, residences were chosen according to the maps and lists of each PSU. Finally, for the respondent-level sampling, one member aged 45 years or older was randomly selected from each chosen household to serve as the main respondent. The baseline investigation was conducted between 2011 and 2012 with respondents and their spouses, and follow-up surveys were performed in 2013, 2015, 2018, and 2020. Over time, some respondents were lost to follow-up, and new participants were selected using the same methods as before. Data regarding demographic information and other characteristics—including health and functional status, economic situation, work, and retirement status—were collected via face-to-face computer-assisted personal interviews. CHARLS implemented effective quality control measures—including interviewer training, the use of standardized questionnaires, a computer-assisted interviewing system, ongoing supervision, and data verification—to control interrater variability among interviewers. Detailed descriptions of the objectives, design, sample, and questionnaires of CHARLS have been reported in previous cohort studies [27].

**Figure 1:**
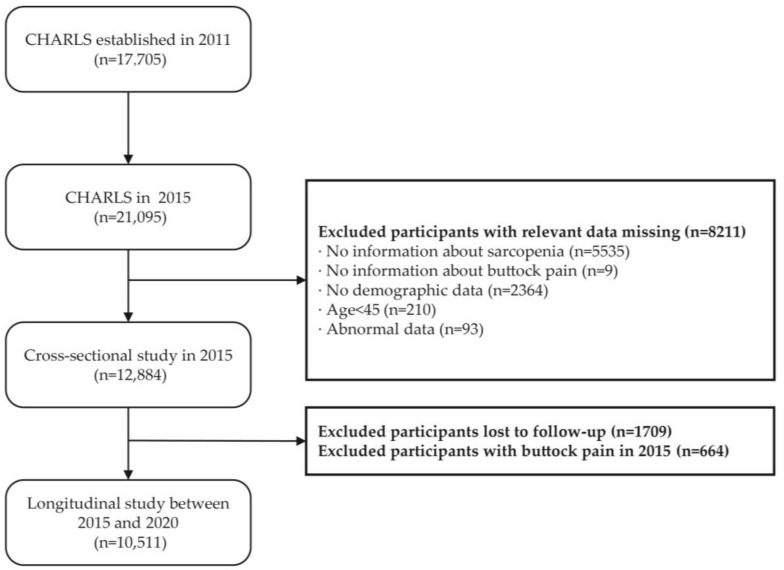
Flowchart of the participant selection process.

In this study, we conducted cross-sectional and longitudinal analyses using data from CHARLS waves in 2015 and 2020. Inclusion criteria were: (1) individuals aged 45 years or older in the 2015 CHARLS sample, and (2) participants who participated in the physical examination and had available height and weight measurements. Exclusion criteria included: (1) missing data on muscle mass or other sarcopenia-related variables in the 2015 survey; (2) missing information on age, sex, height, or weight; and (3) missing other necessary baseline data (including residency, marital status, alcohol consumption, smoking, sleep duration, and walking ability). Figure 1 illustrates the detailed data screening process.

All rounds of the CHARLS survey were approved by the Peking University Biomedical Ethics Committee with the following approval number: IRB00001052-11015. Informed consent was obtained from all respondents who agreed to participate.

### 2.2. Assessment of Sarcopenia Status

The assessment of sarcopenia was conducted in accordance with the 2019 Asian Working Group for Sarcopenia (AWGS) algorithm and 2021 Chinese consensus on the diagnosis and treatment of sarcopenia among the elderly [28,29]. The evaluation consisted of three components: grip strength, physical performance, and appendicular skeletal muscle mass (ASM). Handgrip strength was measured using a dynamometer (Nantong Yuejian Physical Measurement Instrument Co., Ltd., Nantong, China) on both the dominant and non-dominant hands, with a cutoff of <28 kg for males and <18 kg for females to define low grip strength. Physical performance was assessed using the 5-time chair stand test or gait speed test. For the chair stand test, participants were instructed to perform the sit-to-stand action five times consecutively, and the time taken was recorded; a time greater than 12 s was considered indicative of poor physical performance [28,29]. In the gait speed test, participants were asked to walk back and forth on a 2.5 m long course (total 5 m), and a gait speed of less than 1.0 m/s was considered as poor physical performance [28,29]. ASM was estimated using a validated anthropometric equation specific for the Chinese population (ASM = 0.193 × weight + 0.107 × height − 4.157 × sex − 0.037 × age − 2.631, weight in kg, height in m, age in year; sex: 1 for male and 2 for female) [30]. Previous studies have demonstrated strong consistency between the ASM estimated by this model and dual-energy X-ray absorptiometry (DXA) measurements, with a coefficient of determination (R^2^) of 0.90 and a mean difference of −0.025 kg (standard deviation: 1.63 kg) [28,30]. Low muscle mass was evaluated using the sex-specific height-adjusted muscle mass index (i.e., ASM divided by height squared), known as the skeletal muscle index (SMI), with the cutoff defined as the 20th percentile of the parameter [31,32,33]. Weight and height were measured using standard stadiometers and weighing scales (accuracy to 0.1 cm and 0.1 kg, respectively). In this study, an SMI value below 5.35 kg/m^2^ for females and below 7.03 kg/m^2^ for males was considered indicative of low muscle mass.

**Figure 2:**
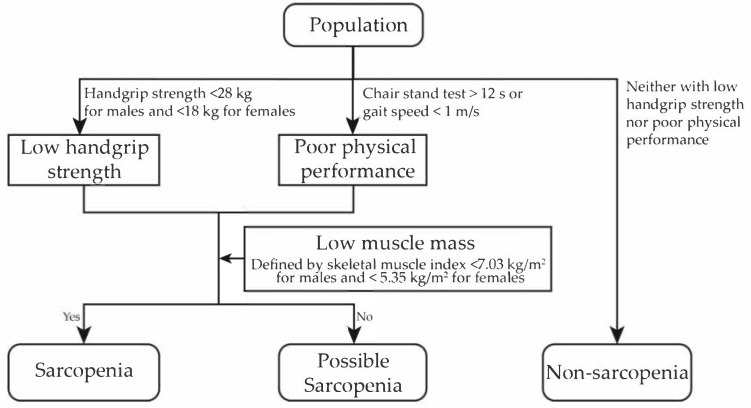
Diagnostic criteria of sarcopenia status and its components in this study.

Referring to the 2019 AWGS algorithm and the 2021 Chinese consensus on the diagnosis and treatment of sarcopenia, sarcopenia status was categorized into non-sarcopenia, possible sarcopenia, and sarcopenia in our study. A subject was diagnosed with sarcopenia if low grip strength or poor physical performance was present in combination with low muscle mass, and if the subject exhibited only low grip strength or poor physical performance without low muscle mass, they were classified as having possible sarcopenia. If both grip strength and physical performance were adequate, the subject was defined as non-sarcopenia (Figure 2). Different from the definition of 2019 AWGS algorithm, where possible sarcopenia is considered a preliminary screening classification that includes individuals with confirmed sarcopenia as well as some individuals without low muscle mass, we defined possible sarcopenia as participants with low muscle strength or poor physical performance but without low muscle mass. The category of severe sarcopenia was not defined separately because the small number of participants in this group would have compromised the statistical power of the analyses.

### 2.3. Assessment of Buttock Pain

In CHARLS, participants were first asked whether they were frequently troubled by body pain. If the response was not “completely no” to this question, they were asked to indicate all areas of pain on a diagram provided by the interviewer, and if they chose buttocks, they were defined as suffering from buttock pain. If participants did not report buttock pain in 2015 but did so at the 2020 follow-up, they were defined as incident buttock pain.

### 2.4. Covariates

Sociodemographic covariates included sex, age, marital status, residence (rural or urban), and educational attainment (primary school or below, secondary school, college, or above). Health-related factors included body mass index (BMI), smoking and drinking habits (yes or no), walking ability, sleep duration, and chronic disease comorbidities. Age was grouped into four categories: 45 to 54 years old, 55 to 64 years old, 65 to 74 years old, and 75 years old or above. For marital status, only participants who were married and cohabiting with their spouse were classified as with spouse, and others were classified as without spouse. BMI was calculated as weight (kg) divided by height squared (m^2^) and was categorized based on the WHO criteria as underweight (BMI < 18.5 kg/m^2^), normal weight (18.5 kg/m^2^ ≤ BMI < 25 kg/m^2^), and overweight or obesity (BMI ≥ 25 kg/m^2^) [34]. Walking ability was divided into four groups: unable to walk, able to walk 100 m, able to walk 1 km, and able to run 1 km. Sleep duration was classified into two groups: less than 7 h and 7 h above. The comorbidities examined in CHARLS included hypertension, dyslipidemia, diabetes, cancer, chronic lung diseases, liver diseases, heart diseases, stroke, kidney diseases, stomach or other digestive diseases, psychiatric disorders, memory-related diseases, and asthma. Participants without any of the above conditions were defined as having no comorbidity. Otherwise, they were considered to have comorbidities.

### 2.5. Statistical Analysis

Continuous variables are presented as the mean ± standard deviation (SD) for normally distributed variables or the median and interquartile range for other continuous variables, while categorical variables are presented as numbers and percentages. The baseline characteristics are summarized according to sarcopenia status, and univariate tests were conducted between groups. For continuous variables with a normal distribution, t-tests or one-way analysis of variance (ANOVA) tests were used; otherwise, the Wilcoxon rank-sum test or Kruskal–Wallis test was applied. For categorical variables, the chi-square test was used. In the cross-sectional analysis, logistic regression models were employed to evaluate the association between sarcopenia status and the prevalence of buttock pain, with the odds ratios (OR) and their 95% confidence intervals (CIs) being calculated. In the longitudinal analysis, to estimate the relationship between baseline sarcopenia status and incident buttock pain, logistic regression models were used to compute ORs and 95% CIs.

Given that the individual components of sarcopenia (including muscle mass, grip strength, and physical performance) might play different roles in the prevalence and incidence of buttock pain, and that there might be sex disparities, subgroup analyses by sex were further performed to explore the relationships between sarcopenia (and its components) and buttock pain.

Multiple logistic regression models were applied in both the cross-sectional and longitudinal analyses: In model 1, sex and age were adjusted; in model 2, sex, age, residence, marital status, educational attainment, BMI category, smoking and drinking habits, comorbidities, sleep duration, and walking ability were adjusted; in model 3, we tried a multivariable model based on variable selection using lasso regression. The variables included in both the cross-sectional and longitudinal analyses are presented in Table S1. Although CHARLS adopts a complex sampling design to ensure national representativeness, the primary objective of this study was to investigate the association between sarcopenia and buttock pain rather than to estimate population-level prevalence or incidence rates. Accordingly, we prioritized internal validity by rigorously adjusting for key confounders (e.g., age, comorbidities) over applying sampling weights for generalizability.

All statistical analyses were performed using R software version 4.2.2 (R Foundation for Statistical Computing). A two-tailed *p*-value < 0.05 was considered statistically significant.

## 3. Results

### 3.1. Baseline Characteristics of the Study Population

A total of 12,884 participants were included in the study. Table 1 summarizes the baseline characteristics of the study population, with a median age of 61 years old (quartile: 53–67 years old) including 6749 females (52.38%). The prevalence of possible sarcopenia and sarcopenia in the study population was 20.11% (2591/12,884) and 9.10% (1172/12,884), respectively. Compared with participants without sarcopenia, those with sarcopenia had a higher proportion of females (56.4% vs. 50.49%), were older (median age of 72 vs. 59 years old), more likely to reside in rural areas (87.88% vs. 73.85%), more likely to be without spouse (31.83% vs. 14.33%); and had a lower educational attainment (89.59% at primary school or below vs. 61.99%), lower BMI (19.45 vs. 23.80 kg/m^2^), a higher proportion with chronic diseases (66.55% vs. 61.86%), higher proportion with less than 7 h of sleep (55.46% vs. 51.73%), and a higher proportion with limitations in walking ability (6.31% vs. 0.72%) (all *p* < 0.05).

### 3.2. Cross-Sectional Analysis of the Association Between Sarcopenia and Buttock Pain

**Figure 3:**
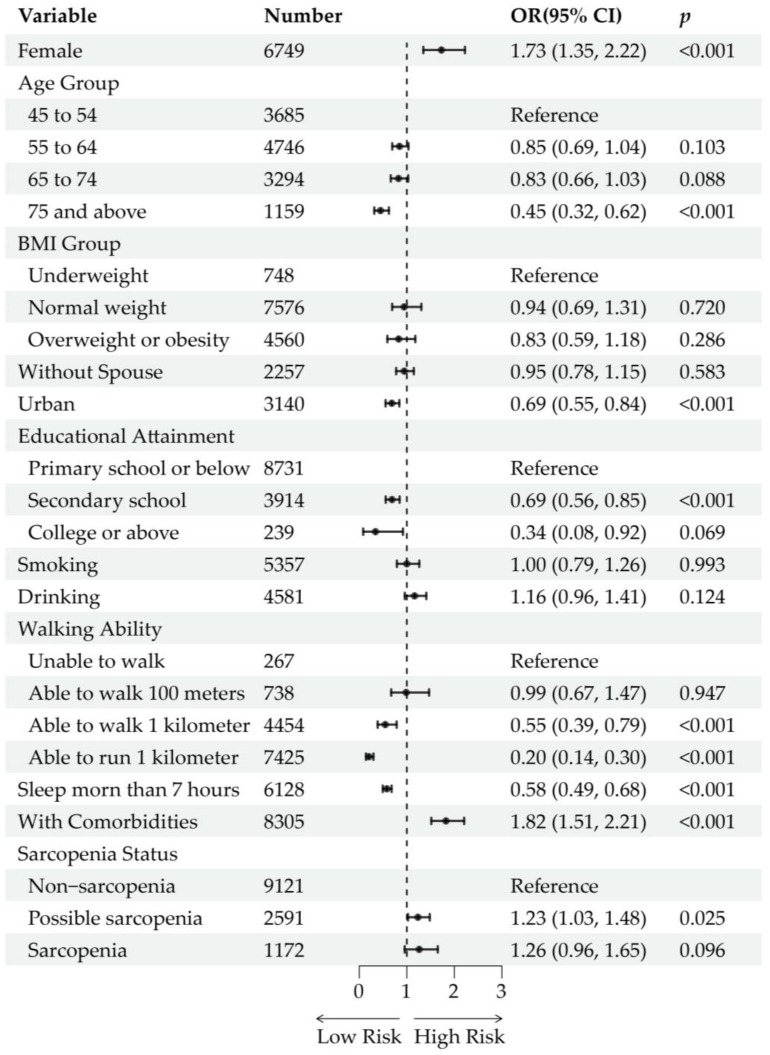
Cross-sectional analysis of the association between sarcopenia status and buttock pain. Forest plots show the odds ratios (ORs) and 95% confidence intervals (CIs) of each variable and level. The ORs are represented by black dots. The horizontal lines depict the 95% CIs.

The overall prevalence of buttock pain in the cross-sectional study sample was 5.94% (765/12,884). For participants with different statuses of sarcopenia, the prevalence of buttock pain was 4.87% (444/9121) in non-sarcopenic participants, 8.45% (219/2591) in those with possible sarcopenia, and 8.70% (102/1172) in those with sarcopenia. After adjustment for age and sex, possible sarcopenia [OR (95% CI): 1.69 (1.41, 2.01), *p* < 0.001] and sarcopenia [OR (95% CI): 1.81 (1.41, 2.31), *p* < 0.001] were significantly associated with the prevalence of buttock pain (Figure S1). However, after further adjustment for sociodemographic and health-related factors, possible sarcopenia [OR (95% CI): 1.23 (1.03, 1.48), *p* = 0.025] remained significantly associated with buttock pain prevalence, whereas sarcopenia [OR (95% CI): 1.26 (0.96, 1.65), *p* = 0.096] did not reach statistical significance (Figure 3). Moreover, following variable selection using lasso regression and subsequent logistic regression analysis, the associations between possible sarcopenia [OR (95% CI): 1.19 (0.98, 1.43), *p* = 0.071] and sarcopenia [OR (95% CI): 1.29 (0.98, 1.69), *p* = 0.071] with buttock pain prevalence were not statistically significant (Figure S2). In the multivariable regression models, increased risk of buttock pain was associated with being female, residing in rural areas, lower educational level, poorer walking ability, sleep duration less than 7 h, and the presence of comorbidities (including liver disease, kidney disease, digestive disease, and memory-related diseases).

### 3.3. Longitudinal Analysis of the Association Between Sarcopenia and Incident Buttock Pain

**Figure 4:**
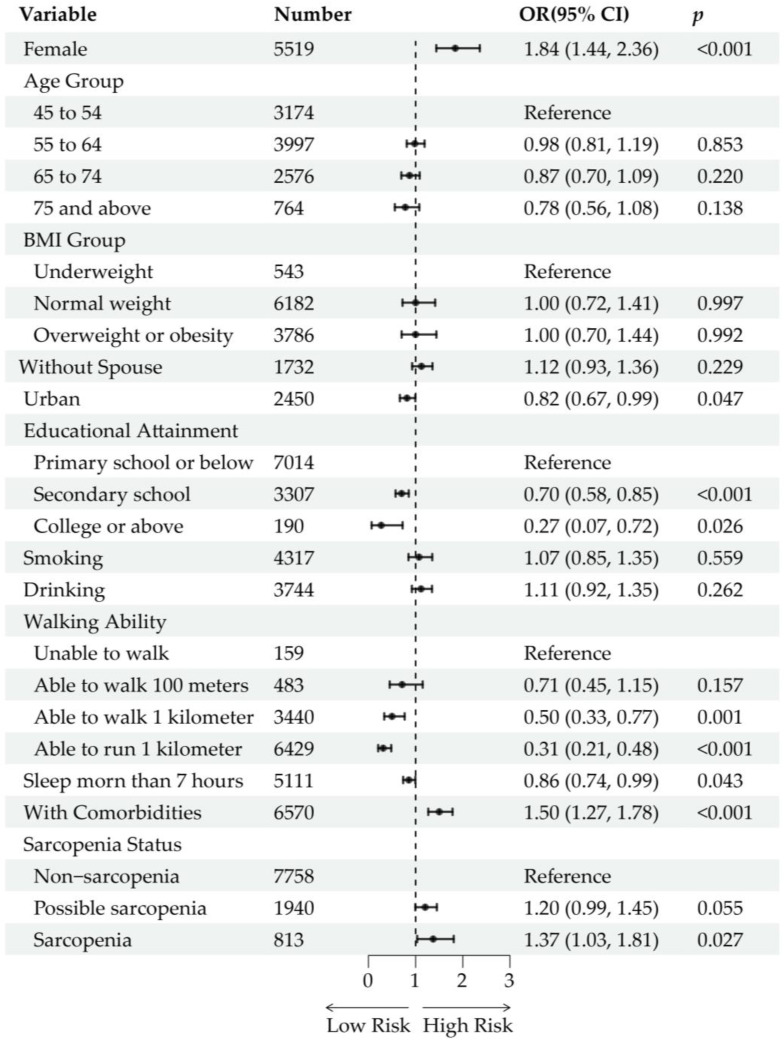
Longitudinal analysis of the association between sarcopenia status and incident buttock pain. Forest plots show the odds ratios (ORs) and 95% confidence intervals (CIs) of each variable and level. The ORs are represented by black dots. The horizontal lines depict the 95% CIs.

Of the 12,884 baseline participants, 1709 (13.26%) were lost to follow-up due to mortality (n = 380), refusal, or other reasons (n = 1329). After excluding participants who had reported buttock pain at baseline (n = 664), 10,511 participants were included in the analysis of incident buttock pain. During the 5-year follow-up, 776 participants (7.38%) developed incident buttock pain. After adjustment for age and sex, possible sarcopenia [OR (95% CI): 1.48 (1.23, 1.77), *p* < 0.001] and sarcopenia [OR (95% CI): 1.66 (1.27, 2.14), *p* < 0.001] were significantly associated with incident buttock pain (Figure S3). With further adjustment for sociodemographic and health-related factors, possible sarcopenia [OR (95% CI): 1.20 (0.99, 1.45), *p* = 0.055] was not significantly associated with incident buttock pain, whereas sarcopenia [OR (95% CI): 1.37 (1.03, 1.81), *p* = 0.027] remained significantly associated (Figure 4). The lasso regression-adjusted model produced similar results, with possible sarcopenia [OR (95% CI): 1.18 (0.97, 1.42), *p* = 0.087] not being significantly associated and sarcopenia [OR (95% CI): 1.36 (1.04, 1.77), *p* = 0.022] being significantly associated with incident buttock pain (Figure S4). In the multivariable regression models, being female, having lower educational attainment, having limited walking ability, and the presence of comorbidities (diabetes, liver disease, heart disease, kidney disease, and digestive disease) were associated with an increased risk of incident buttock pain.

### 3.4. Subgroup Analyses of the Associations Between Sarcopenia, Muscle Characteristics, and Buttock Pain

A total of 12,884 participants were included in the analysis of the associations between muscle characteristics (muscle mass, grip strength, and physical performance) and the prevalence of buttock pain, including 6135 male participants and 6749 female participants (results are shown in Table S2). After adjusting for age and sex, all the muscle characteristics were associated with the prevalence of buttock pain. However, after adjusting for sociodemographic and health-related variables, only physical performance remained significantly associated with buttock pain [Model 2 OR (95% CI): 1.25 (1.05, 1.50), *p* = 0.013; Model 3 OR (95% CI): 1.25 (1.05, 1.50), *p* = 0.014], indicating that poorer physical performance is associated with a higher prevalence of buttock pain. Subgroup analysis by sex revealed that physical performance was still associated with buttock pain prevalence among females [Model 2 OR (95% CI): 1.25 (1.01, 1.55), *p* = 0.037, Model 3 OR (95% CI): 1.25 (1.01, 1.55), *p* = 0.042] but not males.

For the analysis of incident buttock pain, a total of 10,511 participants (4992 male participants and 5519 female participants) were included (Table S3). After adjusting for covariates, low grip strength [Model 2 OR (95% CI): 1.31 (1.07, 1.60), *p* = 0.007, Model 3 OR (95% CI): 1.29 (1.05, 1.57), *p* = 0.013] and sarcopenia were associated with incident buttock pain, with subgroup analysis indicating that the association between grip strength and incident buttock pain was significant only in men [Model 2 OR (95% CI): 1.62 (1.13, 2.27), *p* = 0.007, Model 3 OR (95% CI): 1.61 (1.13, 2.26), *p* = 0.008]. In sex-stratified analyses, neither the association between sarcopenia and incident buttock pain reached statistical significance in males or females. Poor physical performance and low muscle mass were not significantly associated with incident buttock pain.

## 4. Discussion

In this study, we found that sarcopenia was not significantly associated with the prevalence of buttock pain, but it was a risk factor for incident buttock pain. In females, poorer physical performance was related to an increased prevalence of buttock pain, while in males, low grip strength is associated with an increased risk of incident buttock pain.

Sarcopenia is defined by the combination of low muscle mass, low muscle strength, and poor physical performance. Before analyzing sarcopenia as a whole, we first examined the relationships between its individual components (muscle mass, grip strength, and physical performance) and buttock pain. Regarding muscle mass, our study found no significant association between low muscle mass and either the prevalence or incidence of buttock pain. Previous studies on the association of muscle and buttock pain are sparse, possibly because the anatomical locations of the hip and buttock are in close proximity, leading many cases of buttock pain to be classified as hip pain and thus causing them to be rarely described separately [16]. Some studies have reported that patients with hip osteoarthritis have significantly lower lower limb muscle mass compared with controls [19] and that the volumes of the gluteus maximus and gluteus medius are smaller in these patients [18]. However, other studies have suggested that there is no significant difference in the volumes of the gluteus maximus, gluteus medius, tensor fasciae latae, and quadriceps between hip pain patients and controls, nor between the affected and unaffected sides in patients [22]. Another study found no significant difference in the volume of hip abductor muscles in women with lateral hip pain compared with controls [23]. Our finding of no significant association between low muscle mass and buttock pain may be attributed to two possible factors. On the one hand, in our study, muscle mass is evaluated through anthropometric equation, which is indirect and may be influenced by somatotype particularly in obese participants, potentially failing to accurately identify sarcopenic obesity. Moreover, the assessment algorithms and cutoff values for low muscle mass remain controversial, and our chosen method may not perfectly reflect actual muscle mass. On the other hand, it is also possible that muscle mass alone is not strongly associated with buttock pain. Muscle function depends not only on mass but also on muscle composition, strength, and neuromuscular control. A reduction in muscle mass that does not result in a concomitant decline in muscle strength may be insufficient to induce significant functional deficits and thus may not be directly related to the prevalence of buttock pain.

Regarding grip strength, our study found that while grip strength was not significantly associated with the prevalence of buttock pain, it was associated with incident buttock pain, particularly in male participants. Few studies have examined the relationship between grip strength and buttock pain. Jia et al., using bidirectional Mendelian randomization, showed a causal relationship between grip strength and osteoarthritis [21]. However, our results suggest that grip strength was not associated with the prevalence. On the one hand, according to previous studies, grip strength may not adequately reflect lower limb muscle strength. Although grip strength is known to be correlated with overall and lower limb muscle strength [35,36] and is used as a screening tool for sarcopenia, some studies have noted that lower limb strength declines faster with age than upper limb strength [37] and that lower limb strength is more closely associated with functional performance compared with grip strength [38]. Therefore, while grip strength is a simple and feasible measure, it may not accurately reflect lower limb muscle function. On the other hand, the negative findings in the cross-sectional analysis might indicate that grip strength (or lower limb strength) is not directly linked to buttock pain symptoms. Muscle function depends not only on static strength but also on muscle activation patterns, and low muscle strength may not necessarily indicate poor muscle function. Additionally, the impact of strength on joint structure may be cumulative and delayed, which may explain why the association was only observed longitudinally, where a decline in grip strength was associated with an increased risk of incident buttock pain. Previous studies have also suggested that muscle strength plays a role in the progression of joint disease. For example, Tateuchi et al. demonstrated that reductions in the strength of the gluteus maximus and deep external rotators led to increased stress in the anterior hip region via musculoskeletal modeling [39]. Another study using cluster analysis found that muscle strength balance, rather than absolute muscle strength, was associated with the progression of hip osteoarthritis, with relative weakness in hip flexors and internal rotators being linked to radiographic progression [40]. These studies suggest that it is important to consider not only overall muscle strength but also local muscle strength balance. Interestingly, in the sex-stratified subgroup analysis, the association between low grip strength and the risk of developing buttock pain was observed only in males, but not in females. This sex-specific difference may be attributed to multiple factors, and one possible explanation is the differing trajectories of age-related changes in grip strength between males and females. Previous studies have shown that although grip strength generally declines with age in older adults, the timing and magnitude of decline differ by sex [41]. Males may experience a greater percentage of decline and a faster rate of decrease compared with females [42,43]. This may suggest that male participants with low grip strength at baseline are more likely to experience more severe deterioration in muscle function in subsequent years, potentially leading to an increased risk of buttock pain.

As for physical performance, our study indicates that physical performance is associated with the cross-sectional prevalence of buttock pain, but not with incident buttock pain. Measures such as gait speed or the chair stand test reflect lower limb muscle function and may be influenced by strength, muscle composition, and pain [44,45,46]. The association of physical performance with the prevalence of buttock pain may in part be due to its reflection of the present state of muscle function as well as the impact of pain on performance. Its lack of association with incident pain may be due to the fact that physical performance more accurately reflects current function rather than the trend of functional decline. The association between physical function and the prevalence of buttock pain was observed only in females. We speculate that this phenomenon may be related to a range of biopsychosocial factors, including differences in hormone levels, baseline physical function, and pain perception [47,48,49]. However, current evidence on this topic remains limited, and further research is needed to confirm these findings.

The definition of sarcopenia encompasses low muscle mass, low muscle strength, and poor physical performance, and our analyses suggest that examining any one component in isolation has limited predictive value for buttock pain. Instead, these factors should be considered collectively when assessing the overall status of muscle function and its relationship with pain. Although studies about sarcopenia and musculoskeletal pain remain relatively few, our findings indicate that while sarcopenia is not significantly associated with the prevalence of buttock pain, it is significantly related to incident buttock pain. Poor physical performance is associated with the prevalence of buttock pain, whereas sarcopenia is not, possibly due to the diagnostic criteria for sarcopenia, which require the concomitant presence of low grip strength or poor physical performance along with low muscle mass. This requirement may introduce heterogeneity and reduce sample size, thereby attenuating the observed associations. The association between sarcopenia and incident buttock pain may reflect the cumulative effects of reductions in muscle mass and strength or indicate that sarcopenia may act as an independent risk factor to increase the risk of buttock pain. In subgroup analyses stratified by sex, the lack of a significant association between sarcopenia and incident buttock pain in either males or females may be partly due to reduced sample sizes after stratification. Future studies should further evaluate the relationships among sarcopenia, musculoskeletal pain, and their underlying mechanisms through epidemiological, biomechanical, experimental, and clinical trials.

This study has several innovative aspects. Firstly, to the best of our knowledge, this is the first large-scale longitudinal study in the Chinese population to examine the association between sarcopenia and buttock pain, thereby providing valuable evidence for the prevention and intervention of buttock pain. Secondly, the study systematically analyzed sarcopenia and its components, including muscle mass, grip strength, and physical performance, offering an in-depth interpretation of their respective roles in muscle function. By combining cross-sectional and longitudinal analyses, it revealed the dynamic roles that muscle characteristics play in the onset and progression of buttock pain. Thirdly, several regression models were used to validate the main findings, and robust conclusions were supported by analyses using three different models as well as by sex-stratified subgroup analyses. Finally, our findings suggest sarcopenia screening as a potential preventive measure for buttock pain. Community-based screening and rehabilitation interventions may help alleviate the disease burden of buttock pain.

However, this study also has limitations. Firstly, although as many risk factors as possible were considered in the analysis, some important covariates (such as body fat percentage, type of occupation, and dietary intake) were not included due to missing content in CHARLS. The estimation of muscle mass was based on an empirical equation that is influenced by height and weight; compared with CT or MRI, it does not provide detailed local muscle characteristics, and unlike DXA, it does not offer information on fat mass, thereby limiting its ability to differentiate sarcopenic obesity. Moreover, while prior studies have confirmed that the equation is highly consistent with DXA in muscle mass calculation, there is a lack of research on its accuracy in diagnosing sarcopenia. Sarcopenic obesity has been linked to various clinical outcomes in hip osteoarthritis [50]. A multicenter longitudinal study found that obesity and sarcopenic obesity, rather than sarcopenia alone, were associated with a higher risk of knee osteoarthritis [51]. Data on body fat percentage would help to further elucidate the relationships among BMI, lean muscle mass, muscle function, sarcopenia, and buttock pain. Daily physical activity is closely related to musculoskeletal disorders and muscle health. However, due to a high proportion of missing data in the dataset, it was not included in the present analysis. Future studies could consider incorporating daily activity measures to account for its potential confounding or mediating effects. Secondly, this study lacks data on younger adults. Because buttock or hip pain occurs across all age groups, our findings, which are primarily associated with degenerative changes in older adults, may not be generalizable to other populations. Thirdly, the absence of sampling weights may limit the generalizability of our findings to populations with socioeconomic characteristics that differ from those of our analytical sample. While our study offers insights into the association between buttock pain and muscle characteristics, it was not designed to estimate national disease burden. Future studies using probability-weighted cohorts are needed to determine the population-level relevance of these associations. Lastly, pain in this study was self-reported, which may introduce bias. Future studies with more specific information might better reveal the relationship between sarcopenia and buttock pain.

## 5. Conclusions

Although sarcopenia is not significantly associated with the prevalence of buttock pain, it is a risk factor for incident buttock pain. Poor physical performance is associated with an increased risk of prevalence of buttock pain, especially in females, whereas low grip strength is associated with an increased risk of incident buttock pain, particularly in males. Low muscle mass is not related to the prevalence or incidence of buttock pain. In summary, analyzing muscle mass, strength, or physical performance in isolation has limited predictive value for buttock pain. These factors should be comprehensively considered when evaluating the relationship between muscle characteristics and buttock pain. The longitudinal association between sarcopenia and buttock pain provided new insights of preventive strategies for buttock pain. We recommend integrating sarcopenia screening into geriatric assessments and promoting community-based muscle training programs to mitigate incident buttock pain.

## Figures and Tables

**Table 1:** Baseline characteristics of the participants by sarcopenia status.

Characteristics	Non-Sarcopenia (n = 9121)	Possible Sarcopenia (n = 2591)	Sarcopenia (n = 1172)	*p*
Female, n (%)	4605 (50.49)	1483 (57.24)	661 (56.40)	<0.001
Age [median (Q1, Q3)], years	59.0 (52.0, 64.0)	64.0 (58.0, 71.0)	72.0 (65.0, 78.0)	<0.001
Age Group, n (%)				
45 to 54	3218 (35.28)	424 (16.36)	43 (3.67)	<0.001
55 to 64	3624 (39.73)	895 (34.54)	227 (19.37)	
65 to 74	1926 (21.12)	902 (34.81)	466 (39.76)	
75 and above	353 (3.87)	370 (14.28)	436 (37.20)	
BMI [median (Q1, Q3)], kg/m^2^	23.80 (21.52, 26.31)	24.71 (22.79, 27.12)	19.45 (18.15, 20.57)	<0.001
BMI Group, n (%)				
Underweight	387 (4.24)	0 (0.00)	361 (30.80)	<0.001
Normal weight	5372 (58.90)	1395 (53.84)	809 (69.03)	
Overweight or obesity	3362 (36.86)	1196 (46.16)	2 (0.17)	
Without Spouse, n (%)	1307 (14.33)	577 (22.27)	373 (31.83)	<0.001
Rural, n (%)	6736 (73.85)	1978 (76.34)	1030 (87.88)	<0.001
Educational Attainment, n (%)				
Primary school or below	5654 (61.99)	2027 (78.23)	1050 (89.59)	<0.001
Secondary school	3265 (35.80)	537 (20.73)	112 (9.56)	
College or above	202 (2.21)	27 (1.04)	10 (0.85)	
Smoking, n (%)	3873 (42.46)	989 (38.17)	495 (42.24)	<0.001
Drinking, n (%)	3387 (37.13)	807 (31.15)	387 (33.02)	<0.001
Walking Ability, n (%)				
Unable to walk	66 (0.72)	127 (4.90)	74 (6.31)	<0.001
Able to walk 100 m	292 (3.20)	299 (11.54)	147 (12.54)	
Able to walk 1 km	2741 (30.05)	1158 (44.69)	555 (47.35)	
Able to run 1 km	6022 (66.02)	1007 (38.87)	396 (33.79)	
Sleep less than 7 h, n (%)	4718 (51.73)	1388 (53.57)	650 (55.46)	0.024
With Comorbidities, n (%)	5642 (61.86)	1883 (72.67)	780 (66.55)	<0.001
Handgrip Strength [median (Q1, Q3)], kg	31.75 (26.00, 39.00)	23.75 (17.50, 29.25)	20.00 (15.50, 25.06)	<0.001
Chair Stand Test [median (Q1, Q3)], s	8.16 (6.84, 9.59)	12.68 (10.37, 14.69)	12.15 (9.28, 14.47)	<0.001
Gait Speed [median (Q1, Q3)], m/s	1.67 (1.41, 1.93)	1.34 (1.02, 1.64)	1.27 (0.98, 1.55)	<0.001
Buttock Pain, n(%)	444 (4.87)	219 (8.45)	102 (8.70)	<0.001

Abbreviation: BMI, body mass index; Q1, 25% quartile; Q3 75% quartile.

## Data Availability

The raw data from CHARLS are available for download on the official website upon request (https://charls.charlsdata.com/pages/data/111/zh-cn.html, accessed on 16 October 2024.). The analytical data used in this study are available upon request to the corresponding author.

## Supplementary Materials

The following supporting information can be downloaded at: https://www.mdpi.com/article/10.3390/healthcare13111311/s1, Table S1: Variates included in model 3 in different analyses; Table S2: Subgroup analysis of associations between muscle mass, handgrip strength, physical performance, sarcopenia, and buttock pain prevalence in the cross-sectional study; Table S3: Subgroup analysis of associations between muscle mass, handgrip strength, physical performance, sarcopenia, and buttock pain incidence in the longitudinal study; Figure S1: ORs and 95% CIs of the cross-sectional analysis between sarcopenia status and buttock pain prevalence (model 1); Figure S2: ORs and 95% CIs of the cross-sectional analysis between sarcopenia status and buttock pain prevalence (model 3); Figure S3: ORs and 95% CIs of the longitudinal analysis between sarcopenia status and buttock pain incidence (model 1); Figure S4: ORs and 95% CIs of the longitudinal analysis between sarcopenia status and buttock pain incidence (model 3).

## Abbreviations

The following abbreviations are used in this manuscript:

ASM	Appendicular skeletal muscle mass
AWGS	Asian Working Group for Sarcopenia
BMI	Body mass index
CHARLS	China Health and Retirement Longitudinal Study
DXA	Dual-energy X-ray absorptiometry
PSUs	Primary sampling units
SMI	Skeletal muscle index
