# Supplementary material for: Association Between Sarcopenia and Buttock Pain Among Middle-Aged and Older Chinese People: Evidence from the China Health and Retirement Longitudinal Study
Source: Healthcare (Basel). 2025 May 31;13(11):1311. doi: 10.3390/healthcare13111311 (PMC12154368; doi:10.3390/healthcare13111311)
Supplement: Supplementary file 1 [file healthcare-13-01311-s001.zip › healthcare-3635589-supplementary.pdf]

Supplementary material

Title:

Association between Sarcopenia and buttock pain among middle-aged and older Chinese: Evidence from the China health and retirement longitudinal study

Authors:

Jian Jin, Huibin Long, Huiwen Zhang, Chuanhui Zhang, Jianhao Lin

Corresponding Author:

Jianhao Lin, MD

Arthritis Clinical and Research Center, Peking University People's Hospital, Peking University

linjianhao@pkuph.edu.cn

Table of Contents:

Table S1. Variates included in model3 in different analyses

Table S2. Subgroup analysis of associations between muscle mass, handgrip strength, physical performance, sarcopenia and buttock pain prevalence in the cross-sectional study

Table S3. Subgroup analysis of associations between muscle mass, handgrip strength, physical performance, sarcopenia and buttock pain incidence in the longitudinal study

Figure S1. ORs and 95% CIs of cross-sectional analysis between sarcopenia status and buttock pain prevalence (model 1)

Figure S2. ORs and 95% CIs of cross-sectional analysis between sarcopenia status and buttock pain prevalence (model 3)

Figure S3. ORs and 95% CIs of longitudinal analysis between sarcopenia status and buttock pain incidence (model 1)

Figure S4. ORs and 95% CIs of longitudinal analysis between sarcopenia status and buttock pain incidence (model 3)

Table S1. Variables included in model3 in different analyses

Analyses	Variables included in model3
**Cross-sectional study of buttock pain and sarcopenia**	
Overall	Sex, Age Group, Educational Attainment, BMI Group, Residence, Marital Status, Drinking Habits, Dyslipidemia, Diabetes, Cancer, Chronic Lung Diseases, Heart Diseases, Stroke, Psychiatric Disorders, Asthma, Sleep Duration, Walking Ability, Liver Diseases, Kidney Diseases, Digestive Diseases, Memory-related Diseases, Sarcopenia Status
**Longitudinal study of buttock pain and sarcopenia**	
Overall	Sex, Age Group, Educational Attainment, Residence, Marital Status, Diabetes, Cancer, Chronic Lung Diseases, Heart Diseases, Psychiatric Disorders, Asthma, Sleep Duration, Walking Ability, Liver Diseases, Kidney Diseases, Digestive Diseases, Memory-related Diseases, Sarcopenia Status
**Cross-sectional study of buttock pain and components of sarcopenia**	
Overall	Sex, Age Group, Educational Attainment, BMI Group, Residence, Marital Status, Drinking Habits, Dyslipidemia, Diabetes, Cancer, Chronic Lung Diseases, Heart Diseases, Stroke, Psychiatric Disorders, Asthma, Sleep Duration, Walking Ability, Liver Diseases, Kidney Diseases, Digestive Diseases, Memory-related Diseases, Sarcopenia Status
Male	Age Group, Educational Attainment, BMI Group, Residence, Marital Status, Drinking Habits, Chronic Lung Diseases, Heart Diseases, Psychiatric Disorders, Asthma, Sleep Duration, Walking Ability, Liver Diseases, Kidney Diseases, Digestive Diseases, Memory-related Diseases, Sarcopenia Status
Female	Age Group, Educational Attainment, Residence, Drinking Habits, Dyslipidemia, Diabetes, Cancer, Chronic Lung Diseases, Heart Diseases, Stroke, Asthma, Sleep Duration, Walking Ability, Liver Diseases, Kidney Diseases, Digestive Diseases, Memory-related Diseases, Sarcopenia Status
**Longitudinal study of buttock pain and components of sarcopenia**	
Overall	Sex, Age Group, Educational Attainment, Residence, Marital Status, Diabetes, Cancer, Chronic Lung Diseases, Heart Diseases, Psychiatric Disorders, Asthma, Sleep Duration, Walking Ability, Liver Diseases, Kidney Diseases, Digestive Diseases, Memory-related Diseases, Sarcopenia Status
Male	Age Group, Educational Attainment, Residence, Marital Status, Cancer, Psychiatric Disorders, Walking Ability, Liver Diseases, Kidney Diseases, Digestive Diseases, Memory-related Diseases, Sarcopenia Status
Female	Age Group, Educational Attainment, BMI Group, Residence, Marital Status, Smoking Habits, Drinking Habits, Diabetes, Chronic Lung Diseases, Heart Diseases, Stroke, Psychiatric Disorders, Asthma, Sleep Duration, Walking Ability, Liver Diseases, Kidney Diseases, Digestive Diseases, Memory-related Diseases, Sarcopenia Status

Table S2. Subgroup analysis of associations between muscle mass, handgrip strength, physical performance, sarcopenia and buttock pain prevalence in the cross-sectional study

**Muscle Characteristics**	**Subgroup**	**OR (95% CI)**		
		**Model1**	**Model2**	**Model3**
Low muscle mass	Overall	1.22 (1.02, 1.47)*	1.20 (0.95, 1.50)	1.21 (0.96, 1.52)
	Male	1.42 (1.03, 1.95)*	1.18 (0.79, 1.74)	1.20 (0.80, 1.77)
	Female	1.14 (0.91, 1.42)	1.21 (0.91, 1.60)	1.14 (0.90, 1.44)
Low handgrip strength	Overall	1.59 (1.31, 1.91)*	1.17 (0.96, 1.42)	1.12 (0.92, 1.36)
	Male	1.67 (1.18, 2.32)*	1.13 (0.79, 1.60)	1.10 (0.77, 1.55)
	Female	1.55 (1.24, 1.94)*	1.18 (0.93, 1.49)	1.12 (0.88, 1.42)
Low physical performance	Overall	1.73 (1.46, 2.05)*	1.25 (1.05, 1.50)*	1.25 (1.05, 1.50)*
	Male	1.80 (1.30, 2.45)*	1.26 (0.90, 1.76)	1.25 (0.88, 1.75)
	Female	1.71 (1.39, 2.09)*	1.25 (1.01, 1.55)*	1.25 (1.01, 1.55)*
Possible sarcopenia	Overall	1.69 (1.41, 2.01)*	1.23 (1.03, 1.48)*	1.19 (0.98, 1.43)
	Male	1.63 (1.17, 2.24)*	1.20 (0.85, 1.69)	1.17 (0.82, 1.66)
	Female	1.71 (1.39, 2.10)*	1.25 (1.00, 1.55)*	1.19 (0.95, 1.49)
Sarcopenia	Overall	1.81 (1.41, 2.31)*	1.26 (0.96, 1.65)	1.29 (0.98, 1.69)
	Male	1.86 (1.17, 2.86)*	1.08 (0.65, 1.74)	1.08 (0.65, 1.75)
	Female	1.80 (1.33, 2.41)*	1.36 (0.97, 1.88)	1.37 (1.00, 1.87)*

* $p < 0.05$

Table S3. Subgroup analysis of associations between muscle mass, handgrip strength, physical performance, sarcopenia and buttock pain incidence in the longitudinal study

**Muscle Characteristics**	**Subgroup**	**OR (95% CI)**		
		**Model1**	**Model2**	**Model3**
Low muscle mass	Overall	1.06 (0.87, 1.27)	1.06 (0.84, 1.34)	1.06 (0.87, 1.29)
	Male	1.24 (0.89, 1.71)	1.03 (0.67, 1.53)	1.14 (0.81, 1.59)
	Female	0.97 (0.77, 1.23)	1.08 (0.81, 1.44)	1.08 (0.81, 1.44)
Low handgrip strength	Overall	1.56 (1.28, 1.90)*	1.31 (1.07, 1.60)*	1.29 (1.05, 1.57)*
	Male	2.06 (1.46, 2.87)*	1.62 (1.13, 2.27)*	1.61 (1.13, 2.26)*
	Female	1.38 (1.08, 1.74)*	1.17 (0.91, 1.50)	1.16 (0.90, 1.48)
Low physical performance	Overall	1.36 (1.13, 1.62)*	1.10 (0.91, 1.32)	1.09 (0.90, 1.32)
	Male	1.56 (1.10, 2.17)*	1.25 (0.87, 1.75)	1.19 (0.83, 1.68)
	Female	1.28 (1.03, 1.59)*	1.04 (0.83, 1.31)	1.05 (0.84, 1.32)
Possible sarcopenia	Overall	1.48 (1.23, 1.77)*	1.20 (0.99, 1.45)	1.18 (0.97, 1.42)
	Male	1.72 (1.23, 2.37)*	1.42 (1.00, 1.98)*	1.35 (0.95, 1.89)
	Female	1.38 (1.11, 1.71)*	1.11 (0.89, 1.39)	1.09 (0.87, 1.37)
Sarcopenia	Overall	1.66 (1.27, 2.14)*	1.37 (1.03, 1.81)*	1.36 (1.04, 1.77)*
	Male	2.17 (1.37, 3.35)*	1.47 (0.89, 2.36)	1.58 (0.99, 2.48)
	Female	1.46 (1.05, 1.99)*	1.32 (0.93, 1.85)	1.36 (0.96, 1.91)

* $p < 0.05$

Figure S1. ORs and 95% CIs of cross-sectional analysis between sarcopenia status and buttock pain prevalence (model 1)

Variable	Number	OR(95% CI)	*p*
Female	6749	2.19 (1.87, 2.58)	<0.001
Age Group			
45 to 54	3685	Reference	
55 to 64	4746	1.07 (0.88, 1.30)	0.488
65 to 74	3294	1.33 (1.08, 1.63)	0.007
75 and above	1159	0.82 (0.59, 1.13)	0.230
Sarcopenia Status			
Non−sarcopenia	9121	Reference	
Possible sarcopenia	2591	1.69 (1.41, 2.01)	<0.001
Sarcopenia	1172	1.81 (1.41, 2.31)	<0.001

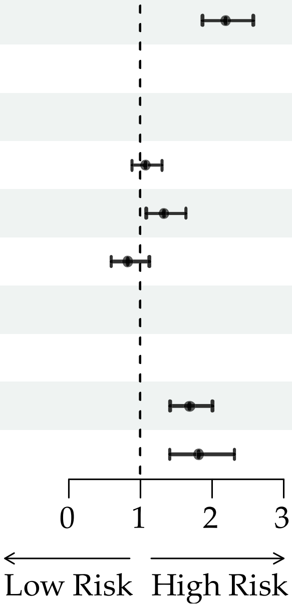

The forest plot shows odd ratios and 95%CIs for buttock pain prevalence after adjusting for sex, age, and sarcopenia status.

Figure S2. ORs and 95% CIs of cross-sectional analysis between sarcopenia status and buttock pain prevalence (model 3)

Variable	Number	OR(95% CI)	*p*
Female	6749	1.75 (1.44, 2.14)	<0.001
Age Group			
45 to 54	3685	Reference	
55 to 64	4746	0.85 (0.70, 1.05)	0.129
65 to 74	3294	0.86 (0.69, 1.08)	0.194
75 and above	1159	0.50 (0.36, 0.71)	<0.001
BMI Group			
Underweight	748	Reference	
Normal weight	7576	1.02 (0.75, 1.42)	0.885
Overweight or obesity	4560	0.94 (0.67, 1.34)	0.733
Married	2257	0.93 (0.76, 1.14)	0.502
Urban	3140	0.68 (0.54, 0.84)	<0.001
Educational Attainment			
Primary school or below	8731	Reference	
Secondary school	3914	0.70 (0.56, 0.86)	0.001
College or above	239	0.35 (0.08, 0.94)	0.073
Drinking	4581	1.12 (0.92, 1.36)	0.253
Walking Ability			
Unable to walk	267	Reference	
Able to walk 100 meters	738	0.96 (0.65, 1.44)	0.826
Able to walk 1 kilometer	4454	0.56 (0.39, 0.82)	0.002
Able to run 1 kilometer	7425	0.23 (0.15, 0.34)	<0.001
Sleep morn than 7 hours	6128	0.64 (0.54, 0.75)	<0.001
Dyslipidemia	1839	1.14 (0.91, 1.40)	0.243
Diabetes	1153	1.09 (0.85, 1.39)	0.490
Cancer	156	0.76 (0.37, 1.40)	0.411
Chronic Lung Diseases	1504	1.23 (0.98, 1.54)	0.068
Liver Diseases	555	1.40 (1.03, 1.88)	0.027
Heart Diseases	2008	1.19 (0.98, 1.45)	0.072
Stroke	296	1.23 (0.80, 1.82)	0.335
Kidney Diseases	935	1.72 (1.37, 2.16)	<0.001
Digestive Diseases	3255	2.03 (1.73, 2.38)	<0.001
Psychiatric Disorders	161	1.03 (0.60, 1.69)	0.913
Memory−related Diseases	229	1.56 (1.02, 2.32)	0.035
Asthma	630	1.30 (0.95, 1.75)	0.093
Sarcopenia Status			
Non−sarcopenia	9121	Reference	
Possible sarcopenia	2591	1.19 (0.98, 1.43)	0.071
Sarcopenia	1172	1.29 (0.98, 1.69)	0.071

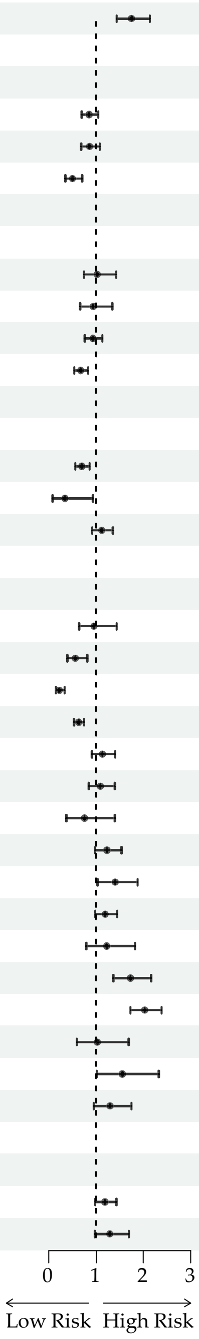

The forest plot shows odd ratios and 95%CIs for buttock pain prevalence after adjusting for sex, age group, educational attainment, BMI group, residence, marital status, drinking habits, dyslipidemia, diabetes, cancer, chronic lung diseases, heart diseases, stroke, psychiatric disorders, asthma, sleep duration, walking ability, liver diseases, kidney diseases, digestive diseases, memory-related diseases, sarcopenia status

.

Figure S3. ORs and 95% CIs of longitudinal analysis between sarcopenia status and buttock pain incidence (model 1)

Variable	Number	OR(95% CI)	*p*
Female	5519	2.08 (1.78, 2.44)	<0.001
Age Group			
45 to 54	3174	Reference	
55 to 64	3997	1.13 (0.94, 1.37)	0.188
65 to 74	2576	1.19 (0.96, 1.47)	0.104
75 and above	764	1.19 (0.87, 1.62)	0.263
Sarcopenia Status			
Non−sarcopenia	7758	Reference	
Possible sarcopenia	1940	1.48 (1.23, 1.77)	<0.001
Sarcopenia	813	1.66 (1.27, 2.14)	<0.001

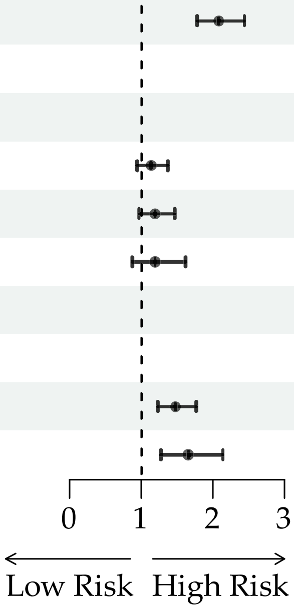

The forest plot shows odd ratios and 95%CIs for buttock pain incidence after adjusting for sex, age, and sarcopenia status.

Figure S4. ORs and 95% CIs of longitudinal analysis between sarcopenia status and buttock pain incidence (model 3)

Variable	Number	OR(95% CI)	*p*
Female	5519	1.68 (1.42, 1.99)	<0.001
Age Group			
45 to 54	3174	Reference	
55 to 64	3997	0.99 (0.81, 1.20)	0.881
65 to 74	2576	0.90 (0.72, 1.12)	0.331
75 and above	764	0.83 (0.59, 1.15)	0.269
Married	1732	1.12 (0.93, 1.36)	0.234
Urban	2450	0.81 (0.66, 0.98)	0.036
Educational Attainment			
Primary school or below	7014	Reference	
Secondary school	3307	0.70 (0.57, 0.85)	<0.001
College or above	190	0.27 (0.07, 0.72)	0.026
Walking Ability			
Unable to walk	159	Reference	
Able to walk 100 meters	483	0.66 (0.42, 1.08)	0.090
Able to walk 1 kilometer	3440	0.48 (0.32, 0.74)	<0.001
Able to run 1 kilometer	6429	0.31 (0.21, 0.49)	<0.001
Sleep morn than 7 hours	5111	0.90 (0.77, 1.05)	0.173
Diabetes	879	1.30 (1.01, 1.64)	0.036
Cancer	113	1.38 (0.73, 2.41)	0.283
Chronic Lung Diseases	1094	1.06 (0.83, 1.36)	0.620
Liver Diseases	407	1.43 (1.02, 1.96)	0.033
Heart Diseases	1448	1.23 (1.00, 1.50)	0.043
Kidney Diseases	668	1.64 (1.26, 2.10)	<0.001
Digestive Diseases	2542	1.56 (1.32, 1.83)	<0.001
Psychiatric Disorders	110	1.73 (0.99, 2.87)	0.041
Memory−related Diseases	149	1.51 (0.91, 2.40)	0.092
Asthma	424	1.08 (0.74, 1.53)	0.692
Sarcopenia Status			
Non−sarcopenia	7758	Reference	
Possible sarcopenia	1940	1.18 (0.97, 1.42)	0.087
Sarcopenia	813	1.36 (1.04, 1.77)	0.022

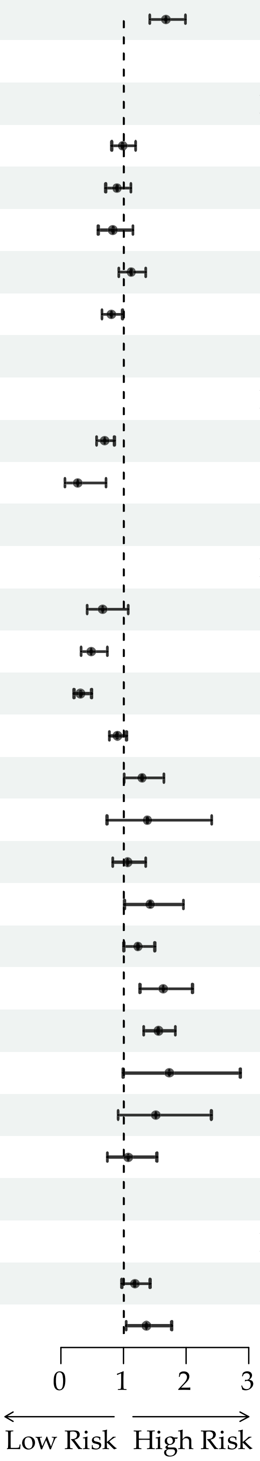

The forest plot shows odd ratios and 95%CIs for buttock pain incidence after adjusting for sex, age group, educational attainment, residence, marital status, diabetes, cancer, chronic lung diseases, heart diseases, psychiatric disorders, asthma, sleep duration, walking ability, liver diseases, kidney diseases, digestive diseases, memory-related diseases, sarcopenia status
